# A denoising framework for 3D and 2D imaging techniques based on photon detection statistics

**DOI:** 10.1038/s41598-023-27852-5

**Published:** 2023-01-24

**Authors:** Vineela Chandra Dodda, Lakshmi Kuruguntla, Karthikeyan Elumalai, Sunil Chinnadurai, John T Sheridan, Inbarasan Muniraj

**Affiliations:** 1 Department of Electronics and Communication Engineering, School of Engineering and Applied Sciences, SRM University AP, Mangalagiri, Andhra Pradesh 522240 India; 2 School of Electrical and Electronic Engineering, College of Architecture and Engineering, University College Dublin, Belfield, Dublin 4 Ireland; 3 LiFE Laboratory, Department of Electronics and Communication Engineering, Alliance College of Engineering and Design, Alliance University, Bengaluru, Karnataka 562106 India

**Keywords:** Electrical and electronic engineering, Imaging and sensing

## Abstract

A method to capture three-dimensional (3D) objects image data under extremely low light level conditions, also known as Photon Counting Imaging (PCI), was reported. It is demonstrated that by combining a PCI system with computational integral imaging algorithms, a 3D scene reconstruction and recognition is possible. The resulting reconstructed 3D images often look degraded (due to the limited number of photons detected in a scene) and they, therefore, require the application of superior image restoration techniques to improve object recognition. Recently, Deep Learning (DL) frameworks have been shown to perform well when used for denoising processes. In this paper, for the first time, a fully unsupervised network (i.e., U-Net) is proposed to denoise the photon counted 3D sectional images. In conjunction with classical U-Net architecture, a skip block is used to extract meaningful patterns from the photons counted 3D images. The encoder and decoder blocks in the U-Net are connected with skip blocks in a symmetric manner. It is demonstrated that the proposed DL network performs better, in terms of peak signal-to-noise ratio, in comparison with the classical TV denoising algorithm.

## Introduction

Auto stereoscopic (i.e., glasses-free) 3D imaging and display techniques have numerous applications in numerous research fields e.g., biomedical, remote sensing, manufacturing, autonomous driving, and augmented reality (AR), to name a few^1–3^. Integral Imaging (II) is one of the techniques that captures a 3D object under incoherent light source and displays a reconstructed 3D scene which can be viewed without the use of special eye wear i.e., 3D glasses^4,5^. In principle, a multiple perspective of a 3D object must be recorded to reconstruct and display a 3D scene. For this purpose, various approaches have been demonstrated in the literature^6^. Owing to the simplified nature of the image capturing process, II has been widely applied^1–3^. To note, II was either combined with existing optical imaging systems or applied directly for auto-stereoscopic 3D imaging applications^6^. For instance, in^7^, an AR based navigation system was demonstrated for an *in-vivo* bio-imaging application. II system was combined with a conventional microscopy for a novel Light Field Microscope^8^. A method to synthesize a digital hologram using II dataset was demonstrated in^9,10^. Furthermore, imaging 3D objects under the turbid water was also proposed in^11^, to mention a few.

In addition to these, a method of photons detection under extremely dark conditions was combined with II systems, known as Photons Counted Integral Imaging (PCII), for low light 3D object imaging and visualization^12^. Thereafter, such system was examined for various applications such as biological imaging^13^, remote sensing^14^, night vision^15^, object detection^16^, autonomous driving^17^ and data encryption^18^, to cite a few. We note that most of these systems were proposed to demonstrate the feasibility of capturing and displaying 3D images under low light. Therefore, these analyses were typically limited to the single channel or monochromatic imaging. Nevertheless, intuitively, colour perception of a 3D object in such a degraded environment should enable better scene interpretation. For this reason, we have developed a simplified single-channel based colour 3D imaging system^19,20^. Our system consists of a DSLR camera which translates both in horizontal (*x*) and vertical (*y*) directions to capture multiple two-dimensional (2D) images (often known as Elemental Images (EIs)) with different perspective. In one of our previous works^19^, we demonstrated that a 3D scene reconstruction is possible from just $\sim$10 photons/scene. However, a 3D object (visual) recognition was possible only with >1000 photons/scene. It is worth mentioning that the photons collection, in a given time interval by a photo sensor, is purely a random process. As a result of this spatial and temporal randomization, PCIs that are recorded, in general, represents a binary image i.e., either the presence (1) or absence (0) of photons. Accordingly, a 3D scene that are generated using the PCII dominated by impulse-like noises^20^. In such cases, it is ideal to use a denoising filter to remove the excessive noises that are prevalent in an image to preserve the scene as much as possible.

Since first proposed, Deep Learning (DL) frameworks^21,22^ have received considerable attention across all disciplines and also among optical engineers and scientists. For instance, DL was applied for 3D object recognition and classification in very low light illumination conditions^23^ . DL was also been shown to be suppressing noises that occur due to the misalignment of optics in diffraction tomography^24^. Similarly, DL network was proposed to remove the speckle noise from Digital Holographic (DH) imaging dataset. In^25^, authors developed a Denoising Convolutional Neural Network (DNCNN) to remove the speckle noise that occurs during phase measurements in DH imaging system. Whereas in^26^, multi-scale U-net architecture was used together with a customized cost function i.e., weighted combination of mean absolute error (MAE) and edge loss to minimize the noise from DH system. Further in^27^, an attention based CNN was proposed in which a customized cost function was developed using polarization loss to denoise the polarimetric images. In addition to this, in^28^, the authors proposed a combined KullBack Liebler (KL) divergence and Total Variation (TV) regularization as a cost function to enhance the denoising performance of the conventional CNN. Recently, a modified DNCNN was developed to enhance the quality of the reconstructed image of polarimetric 3D imaging under a degraded environment^29^. It is evident from these studies that supervised learning (SL) method was primarily used. It is known that, in general, SL requires a larger labeled clean data to train and test the network. However, such a clean labeled data may not be available and generating (synthetically) larger dataset is a time-consuming task. This process is applicable for PCII systems and therefore sufficiently larger (training) dataset is not available. To overcome such limitations, in this paper, for the first time, we propose to use a method which does not require a labeled dataset. Such a network is known as an end-to-end unsupervised network. In this work, we propose to use U-Net architecture with skip blocks^30^ to denoise the photon counted 3D integral imaging dataset.

## Results

**Figure 1:**
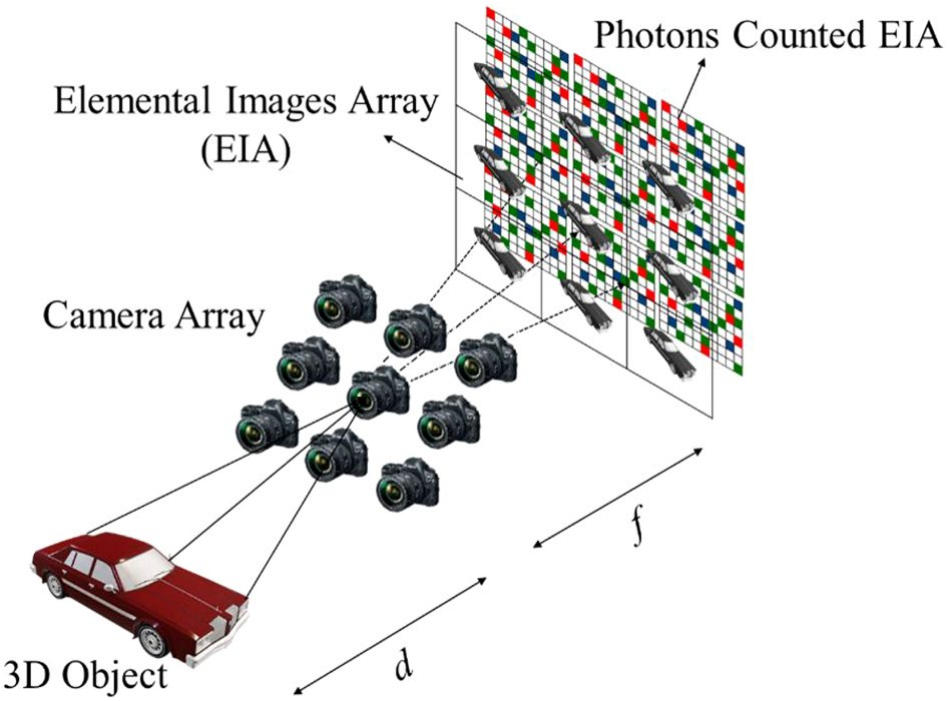
Pickup process of Photons Counted 3D Integral Imaging. Here, camera array depicts the rectangular translation of a single camera on the pick up plane to capture multiple 2D elemental images.

Figure 1 shows the Photons Counted Integral Imaging (PCII) setup. In principle, PCII can be implemented in two steps. *Step 1 (Pickup)*: In this process, a 3D object is being captured (in multiple different perspectives ) by moving the camera both in vertical and horizontal directions. This process results in capturing four-dimensional (4D) light-field data i.e., two spatial dimensions (*x,y*) and two angles in which light rays are measured $(\theta _{ x},\theta _{y} )$^10^. The captured images are known as Elemental Images (EIs) or an Elemental Image Array (EIA). *Step 2 (Reconstruction)*: The recorded 2D EIs are combined to produce a 3D scene. Reconstruction is in fact a reverse process of step 1, therefore by using a ray back propagation technique, a 3D scene can be reconstructed. It is worth to mention the fact that during reconstruction process the objects that were positioned at the same (object) plane combined flawlessly, thus they appear clearly in focus. The objects that were positioned in other planes appear blurry i.e., out of focus. A detailed Bayer patterned based EIA capturing and reconstructing can be found at^19,20^.

In some applications, image reconstruction is shown to be possible with only low scattered photons^31^.This can be done either by employing a physical camera, e.g. EMCCD and sCMOS, to capture a scene at low light conditions^16,31^, or by using the computational approaches^12,32^. In this work, we have used a computational approach, in such cases, a Poisson distribution is used to estimate the photons at any given image^12,29^. Let the total number of photons detected in a normalised elemental image $({\widehat{EI}})$ is $n_{p}$, then by using a Poisson parameter $\lambda$, we can estimate the photons counted image as given below^32^:

1 $$\begin{aligned} P(C_{x} |I_{x})\sim PoissonDistribution(\lambda =n_{p}\times {\widehat{EI}}(x,y)) \end{aligned}$$

We then apply the parametric maximum likelihood estimator (MLE) to the photons counted elemental images to reconstruct the photons counted 3D sectional images^19^:

2 $$\begin{aligned} MLE \{I_{P} ^{Z}\}= \dfrac{1}{n_{p}RS} \sum _{r=1}^{R} \sum _{s=1}^{S} C_{rs}\left( x+r\left( \dfrac{shf_{x}}{MF} \right) ,y + s\left( \dfrac{shf_{x}}{MF}\right) \right) \end{aligned}$$

where *MF* denotes the magnification factor of the imaging system which is given as $$ MF= z/d$$ in which *d* is the distance between pick up grid and image plane, see Fig. 1. Subscripts *r,s* indicate the pick-up location of the elemental image and *p* denotes the photon counted images. $$C_{rs} (.)$$ is the photon counted pixel value of the $$(r,s)$$th elemental image, corresponding to the voxel value $$I_{p}^{z}$$^19^.

### Denoising network

**Figure 2:**
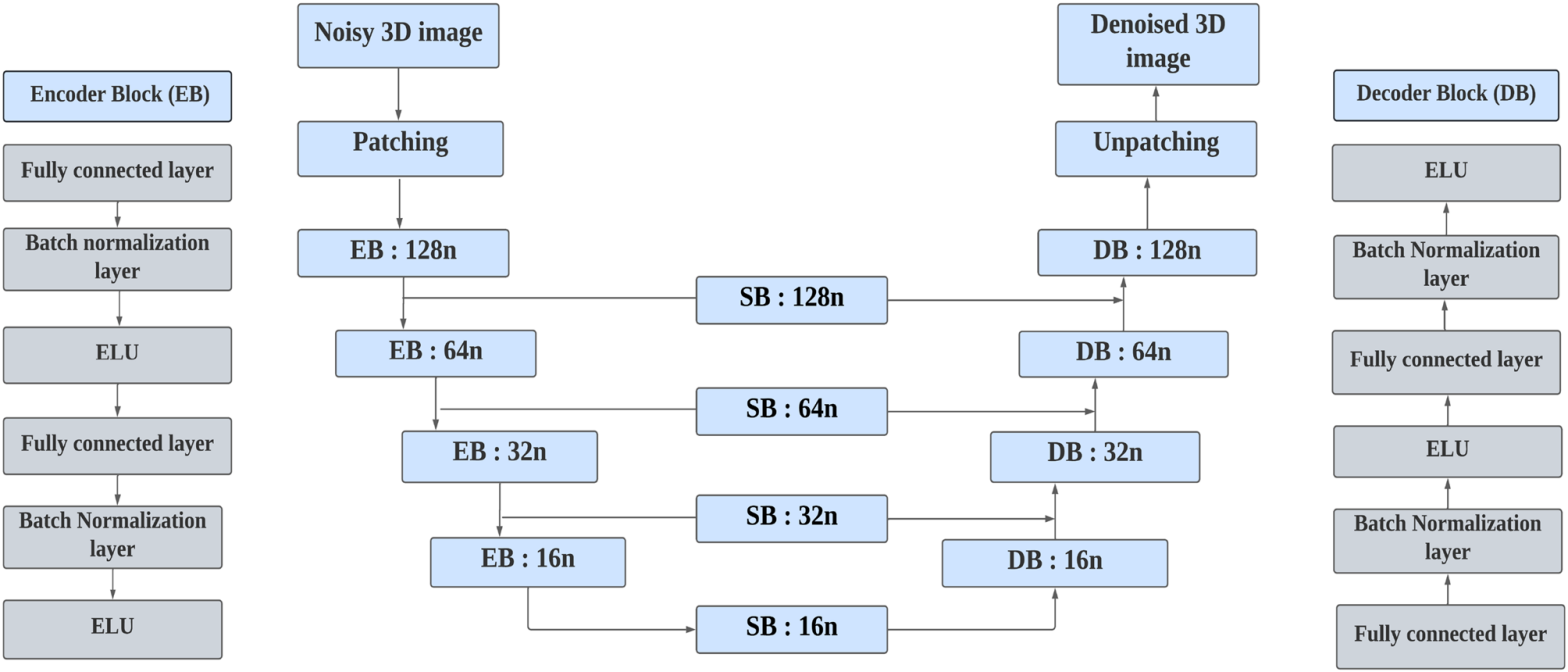
Architecture of the unsupervised denoising network; EB-encoder block, DB-decoder block, and SB-skip block.

In this section, we describe the opted denoising deep learning architecture i.e., U-Net, see Fig. 2. As aforementioned, this is an end-to-end fully unsupervised denoising approach where the noisy photons counted 3D sectional image is fed as an input to the network. This network uses multiple encoder/decoder layers in a symmetric manner to retrieve denoised image with very few training data. Let *x* denote the clean 3D sectional image, *n* be the noise added by the photon counting process to the II system and *y* represent the resulted noisy photons counted 3D sectional images. Mathematically, this is given as:

3 $$\begin{aligned} y = x+n \end{aligned}$$

The objective of image denoising problem is to restore *x* from *y* by attenuating the noise *n*. This process can be given as follows:

4 $$\begin{aligned} {\hat{x}} = {\mathcal {H}}(y;\Theta ) \end{aligned}$$

where ${\hat{x}}$ be the estimated denoised photons counted image, ${\mathcal {H}}(.;\Theta )$ is a parametric function and $\Theta$ are the trainable parameters. The major components in the U-Net are encoder and decoder blocks with skip connection layers^33–35^. In addition to this, skip blocks (SB) are added to the skip connection strategy in U-Net architecture to avoid vanishing gradients problem. These skip blocks and skip connections are designed in encoder and decoder blocks according to the features of the photons counted 3D images. Moreover, this strategy has the advantage of retaining useful image information. The intuitive reason behind adding the skip connections is that the low-level encoder extracts the abstract features which can be lost during the training process of the neural network. To avoid such loss in the features, we add skip connection from the encoder layers to the corresponding decoder layers. In the training process, 3D input image is given in the form of patches to the network. The advantage of applying such a patching technique is to increase the number of training samples such that the image features are learned by the network accurately. In the patching process, the patch window is moved horizontally and vertically to cover the whole image^36^. The patched input image is converted to 1D vector and fed as an input to the network. After removing the noises, we unpatch the 1D vector and convert back to the size of input data.

In the following, we will discuss the details of network blocks used: The encoder block consists of two fully connected layers, two batch normalization layers and two activation layers. The principle behind encoding operation is dimension-reduction thereby extracting the useful image content from the noisy image *y*. The encoding operation is expressed as follows:

5 $$\begin{aligned} d_{e1}=W_{e1} P_{m}+b_{e1} \end{aligned}$$

where $d_{e1}$ is the output from the encoder and $P_{m}$ is the input to the encoder which is generated by the patching process. $W_{e1}$ and $b_{e1}$ are the weight and bias matrices of the *m*th encoding layer in the encoding process. Batch normalization layer is placed to prevent internal covariate shifts^34^. The low-level parameters in the network change the high-level data distributions during training process. This leads to the reduction of network accuracy due to the accumulation of error. However, the batch normalization layer speeds up the network and prevents gradient vanishing problem. The output of the batch normalization layer is passed to Exponential Linear Activation (ELU) function layer^37^, which is given as:

6 $$\begin{aligned} ELU(x)= {\left\{ \begin{array}{ll} x &{} \text {if}\, x\ge 0\\ a(e^{x}-1)&{} \text { otherwise,} \end{array}\right. } \end{aligned}$$

where *a* is hyper-parameter and $a\ge 0$. The advantage of ELU is that it tries to make the mean activations (average activations of neurons in the layer for the given input) close to zero, thus speeding up the network. The output of each encoder block is given as:

7 $$\begin{aligned} {{\hat{d}}_{e1}}=ELU(W_{e1}P_{m}+b_{e1}) \end{aligned}$$

The role of decoder is to reconstruct the photons counted 3D images from the abstract features extracted from the encoder block. The encoder and decoder blocks are symmetrical in structure. The decoder also consists of two dense layers, two batch normalization layers and two activation function layers. The output of decoder block is as follows:

8 $$\begin{aligned} {{\hat{r}}_{dm}}=ELU(W_{dm}K_{m}+b_{dm}) \end{aligned}$$

where $W_{dm}$ and $b_{dm}$ be the weights and biases of the *m*th decoder layer and $K_{m}$ is the input for the *m*th decoder layer. The number of neurons in the encoder block are 128, 64, 32, 16, 8 and vice-versa for the decoder block^34^. The last layer of the network is fully connected layer and reconstructs the output patches into the size of input photons counted image. In the training process, the selection of cost function plays a vital role to obtain optimum parameters i.e., weights and biases. To minimize the cost function, various optimization algorithms were proposed in the literature. For example, gradient descent, stochastic gradient descent, Adaptive Gradient Algorithm (ADAGRAD), Adaptive Moment Estimation (ADAM), to name a few^35,38,39^. In our work, we use the ADAM optimizer to update the parameters $\Theta$. The merits of ADAM include: easy implementation, computationally inexpensive and requires less memory^35^. The ADAM optimizer updates the parameters $(\rho )$ as shown below:

9 $$\begin{aligned} \rho _{(t+1)}=\rho _{t}-\frac{\eta }{\sqrt{({\hat{v}}(t)) +\epsilon }}{\hat{n}}(t), \end{aligned}$$

where ${\hat{v}}(t)$ and ${\hat{n}}(t)$ are bias corrected first and second moments defined as $v_t/1-\beta _2$ and $n_t/1-\beta _1$, respectively. Terms $n_t$ and $v_t$ are exponentially moving averages obtained by using $n_t=\beta _1n_{t-1}+(1-\beta _1)g_t$ and $v_t=\beta _2v_{t-1}+(1-\beta _2)g_t^2$, respectively. We note, $\beta _1$ and $\beta _2$ represents exponential decay rates for the first and second moments with the value of 0.90 and 0.999, respectively. The $g_{t}$ is gradient of cost function with respect to time and $\eta$ is learning rate which is generally set as 0.001^35^. The Mean Squared Error (MSE) is used as the cost function in our training process. The loss is calculated between the input noisy patched photons counted 3D images *P* and output denoised patches obtained from the network as follows:

10 $$\begin{aligned} C(\Theta )= min \parallel \psi (P;\Theta )-P\parallel ^{2} \end{aligned}$$

where $\psi$ denotes the denoising approach, $\psi (P;\Theta )$ are the output patches. During the training process, we adapt an optimization strategy i.e., early stopping. When the cost function of the validation set does not decrease for four consecutive epochs, the denoising network will stop training and save the best parameters^34^. The SB block consists of one fully connected layer, one batch normalization layer, and one ELU layer. The output of *m*th decoder layer is connected to the output of SB block ${\hat{s}}_{dm}$. The final output after each decoder block is ${\hat{r}}_{edl}=\{{\hat{d}}_{e1}, {\hat{s}}_{dm}\}$.

### Experimental results

**Figure 3:**
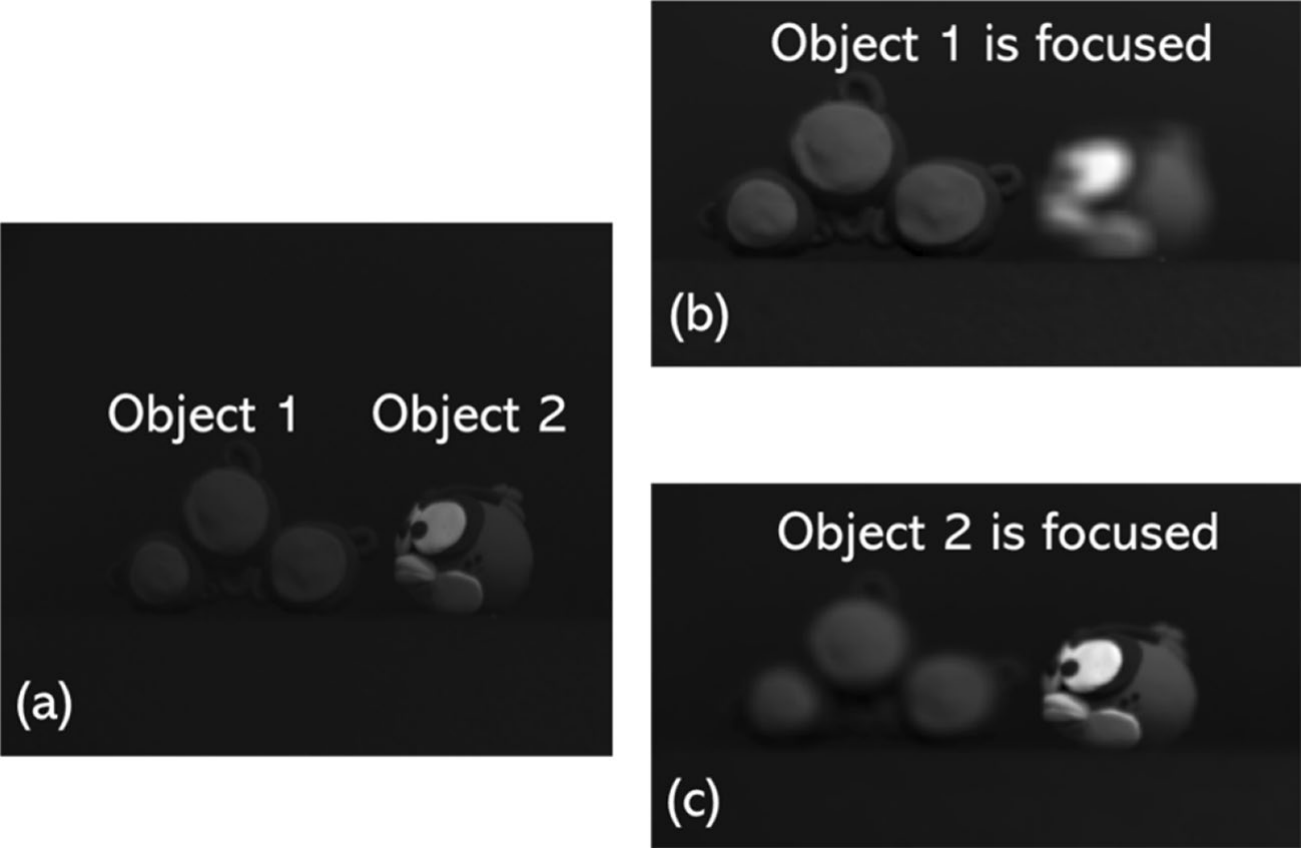
Three-dimensional (3D) objects used in our experiments: (**a**) EI of the 3D scene with Bayer format, (**b**) 3D reconstructed sectional image in which Tri-coloured balls is focused and (**c**) 3D reconstructed sectional image in which Toy bird is focused.

**Figure 4:**
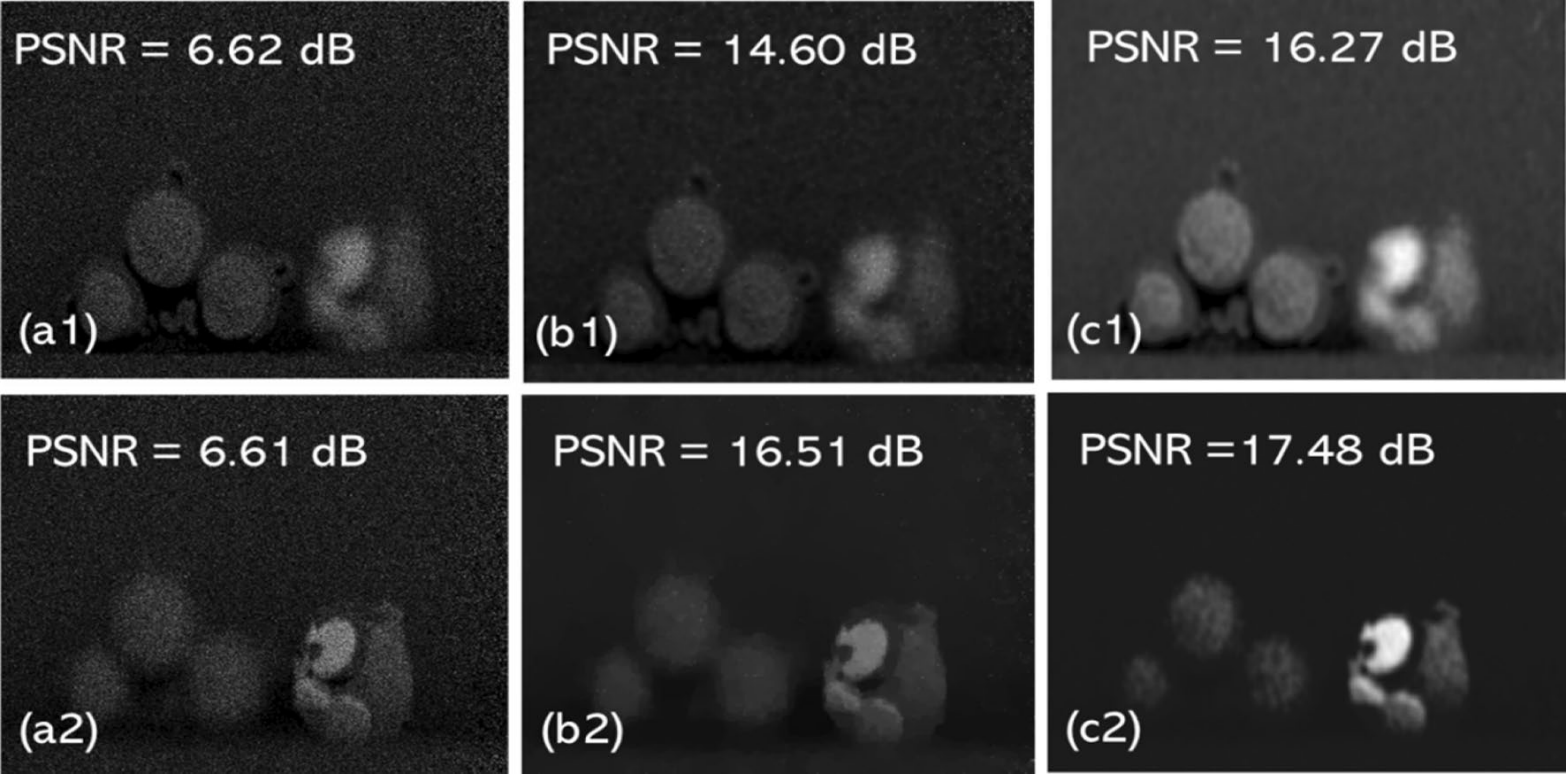
Denoised results: (**a1**, **b1**, **c1**) represents noisy Photon counted 3D sectional image, TV denoised image and result of our proposed denoising method when object 1 is in focus, respectively and (**a2**, **b2**, **c2**) represents the noisy Photon counted 3D sectional image, TV denoised image and result of our proposed denoising method when object 2 is in focus, respectively.

**Figure 5:**
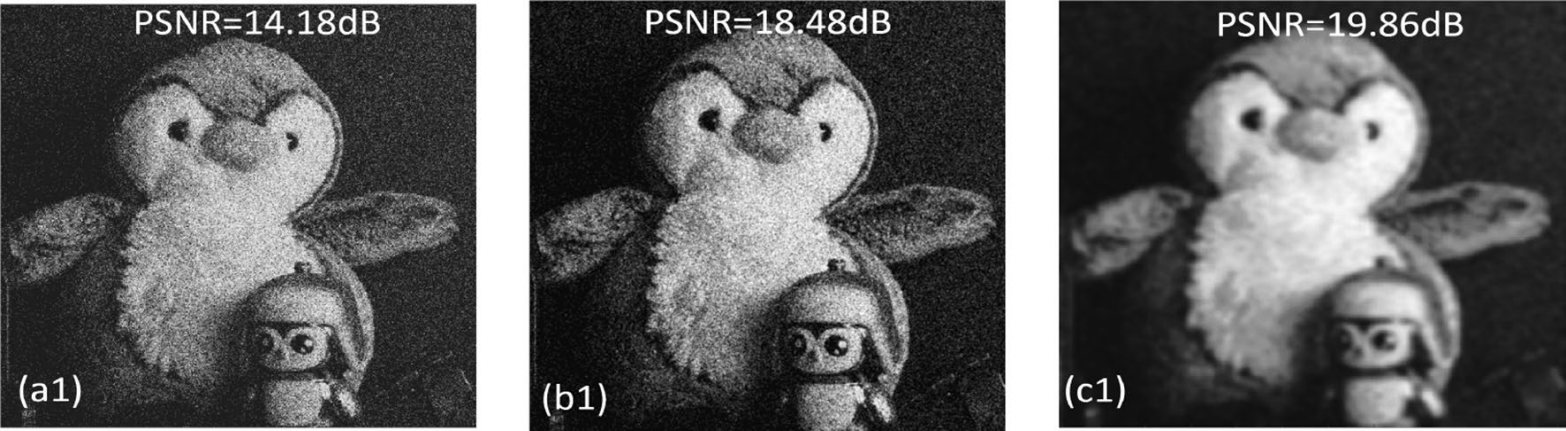
Denoised results: (**a1**), (**b1**), (**c1**) represents noisy QIS image, TV denoised image and result of our proposed denoising method with corresponding PSNR values.

To test the performance of our proposed denoising network, we used $10 \times 10$ elemental images that were captured by shifting our CCD camera with equal separations of 5 mm in both horizontal (H) and vertical (V) directions (see Fig. 1). In our experiments, we used two 3D objects: one is a tri-colored ball known as Object 1 in Fig. 3a and second is a toy bird referred as Object 2 in Fig. 3a. From the pick up grid (i.e., imaging sensor) Object 1 and Object 2 were kept at 370 mm and 500 mm away, respectively. Further, the focal length of our imaging system is 50 mm and the pixel size is $7.4\mu m \times 7.4\mu m$. To note, the elemental images were initially recorded at the size of $1668(H) \times 1668(V)$ but later resized into $422(H) \times 539(V)$ before fed into our proposed DL network. Figure 3a depicts the two 3D objects used in our experiments and Fig. 3b, c shows the clean sectional images i.e., reconstructed 3D depth images via computational approach as described in^19^ without using the photon counting technique. As can be seen from Fig. 3b, c in the reconstructed sectional images only one of the objects is clearly focused (depends on the corresponding depth location) while the other object is off-focused or defocused. Further, to note, the images shown in Fig. 3 were captured as Bayer patterned image (i.e., GRBG format) which has a potential to be converted as a colour (RGB) image using interpolation techniques. Conversion of the Bayer image into a RGB image is out of the scope of this article, therefore not described here. However, readers are recommended to explore^19,20^ where a detailed 3D imaging setup, reconstruction technique and a comparison of various interpolation techniques can be found (Fig. 4). In addition to this 3D dataset, we have also tested the proposed denoising network on a single-photon detector based 2D dataset i.e., Quanta Image sensor (QIS)^40^, for instance see Fig. 5. To reiterate, owing to the stochastic nature of the photons arrival to an imaging sensor, photon shot noise prevails at almost every standard imaging (i.e., CCD and CMOS) system. In such scenarios, denoising becomes a non-trivial task as the captured images are not only dark (see for instance, Figs. 3 and 5) but the noise is camouflaged with the recorded scene which makes it hard to distinguish from the object of interest. In the following, we present our denoising results.

The results are produced by performing simulations on an Intel®  Xeon®  Silver 4216 CPU @2.10 GHz (2 processors) with 256 GB RAM, 64-bit operating system. The software used is Spyder integrated development environment from Anaconda Navigator. It took around 68 s to run the python code and obtain the results. To note, $n_{p}$ = 5000 photons/scene is used to synthesize the photon counted 3D sectional image (PCSI), as described in Eq. (1). Our network is then trained using a single PCSI, which is fed into our DL network in the form of multiple patches. This patching process reduces the time required to create a dataset either by a physical or a synthetic process^41^. Thus, the demand for larger dataset is obviated as the network learns the features only through these patches. We note, our network was tested with various patch sizes to achieve better denoising accuracy. It was estimated, based on our simulations, an 8$\times$8 patch size provides a superior result in terms of peak signal-to-noise ratio (PSNR). It is worth to mention the fact that the selection of smaller patch size gives the finer details while the larger patch size may lose the finer details from an inputted image^42^. To note, we used 20% of the PCSI patches for validation and 60% of patches are allotted for training purpose. The validation loss (val_loss) is continuously monitored, via the model checkpoint callback function, to estimate the optimum model i.e., the loss is minimized. In addition to this, we also used an early stopping criterion that stops the training process when the val_loss is not converging even after at least 5 epochs, in the interest of computational time. In this work, 15 epochs were used with a learning rate of 0.001 to train the network. Further, we also calculated the computational complexity for the proposed denoising architecture and it is estimated the time taken for the classical TV denoising algorithm was 15.09018 s while it takes 2110 s (for both training and testing) to execute the proposed DL network.

In addition to this, to quantitatively evaluate the performance of the opted denoising DL network against the photon counted 3D sectional images and the 2D QIS dataset, we have used two standard image quality metrics: first is the Peak signal-to-noise ratio (PSNR) which is defined as follows:

11 $$\begin{aligned} PSNR= 10.log10 \dfrac{I_{max}^{2}}{MSE} \end{aligned}$$

where $I_{max}$ is the maximum possible pixel intensity value of an image. MSE refers to the mean squared error between the clean image i.e., (Fig. 3b, or c) and the corresponding noisy PCSI (i.e., Fig. 4)^19^. For instance, the PSNR value given in Fig. 4b2 is an estimation between Figs. 3c and 4b2. The second metric, that we used in our simulations to test the performance of the opted denoising DL network, is structural similarity index measure (SSIM), which is given as:

12 $$\begin{aligned} {{\,\textrm{SSIM}\,}}(x,y) = \frac{(2\mu _x\mu _y + C_1) (2 \sigma _{xy} + C_2)}{(\mu _x^2 + \mu _y^2+C_1) (\sigma _x^2 + \sigma _y^2+C_2)} \end{aligned}$$

where $\mu _x, \mu _y, \sigma _x, \sigma _y, and \sigma _{xy}$ represents mean, standard deviation, cross-covariance and $C_1$ and $C_2$ denotes the constant values, respectively^43^. For our proposed DL network, SSIM is calculated as 0.8540 when the Object 1 is in focus (i.e., Fig. 4a1), but when the same 3D sectional image was denoised using the classical TV denoising technique we obtained SSIM of 0.7218. Similarly, when the Object 2 is in focus (i.e., Fig. 4a2) the SSIM value of 0.6445 is achieved with the proposed DL method and 0.5913 with the classical TV denoising method. Furthermore, for the QIS dataset, we estimated SSIM of 0.3222 when our proposed DL method was applied and 0.3205 for the classical TV denoising method.

It is therefore evident from these results that the proposed DL method outperforms the classical TV denoising by maximum of 1.91 dB (for 3D dataset), 1.38 dB (for QIS dataset) in terms of PSNR and by maximum of 0.1322 (for 3D dataset) and 0.0017 (for QIS dataset) in terms of SSIM.

## Conclusion

In summary, we have proposed a deep learning network for denoising the 3D (photons counted three-dimensional integral imaging) and 2D (Quanta Image Sensor) dataset. We demonstrated that it is possible to denoise the low light level imaging dataset using a fully unsupervised network. In this work, encoder and decoder with skip blocks were opted to learn object features from the noisy photon counted 3D sectional images and QIS based images. The patches are selected randomly, covering the whole data, to obtain satisfactory results. As the denoising network does not require clean labels, the method is feasible for use in a wide variety of scenarios. It is therefore planned to extend this investigation by more closely identifying the patching process and parameter tuning in the architecture to achieve better denoised results^42^. This includes examining such network on some classical optical imaging systems that suffer from inevitable noises.

## Data Availability

Data for this paper are not publicly available but shall be provided upon reasonable request to the corresponding author.
